# Supplementary material for: Dopamine and Striatal Neuron Firing Respond to Frequency-Dependent DBS Detected by Microelectrode Arrays in the Rat Model of Parkinson’s Disease
Source: Biosensors (Basel). 2020 Sep 28;10(10):136. doi: 10.3390/bios10100136 (PMC7600337; doi:10.3390/bios10100136)
Supplement: Supplementary file 1 [file biosensors-10-00136-s001.pdf]

biosensors

Supplementary information

Article

Dopamine and Striatal Neuron Firing Respond to Frequency-Dependent DBS Detected by Microelectrode Arrays in the Rat Model of Parkinson's Disease

Guihua Xiao [1,2], Yilin Song [1,2], Yu Zhang [1,2], Yu Xing [1,2], Shengwei Xu [1,2], Mixia Wang [1,2], Junbo Wang [1,2], Deyong Chen [1,2], Jian Chen [1,2] and Xinxia Cai [1,2,*]

1 State Key Laboratory of Transducer Technology, Aerospace Information Research Institute, Chinese Academy of Sciences, Beijing 100190, China; xiaoguihua11@126.com (G.X.); ylsong@mail.ie.ac.cn (Y.S.); zhangyu_diandian@163.com (Y.Z.); xingyu17@mails.ucas.ac.cn (Y.X.); swxu@mail.ie.ac.cn (S.X.); wangmixia@mail.ie.ac.cn (M.W.); jbwang@mail.ie.ac.cn (J.W.); dychen@mail.ie.ac.cn (D.C.); chenjian@mail.ie.ac.cn (J.C.)

2 University of Chinese Academy of Sciences, Beijing 100049, China

* Correspondence: xxcai@mail.ie.ac.cn; Tel.: +86-010-5888-7193

Received: 24 August 2020; Accepted: 21 September 2020; Published: 28 September 2020

1 Supplementary Figures

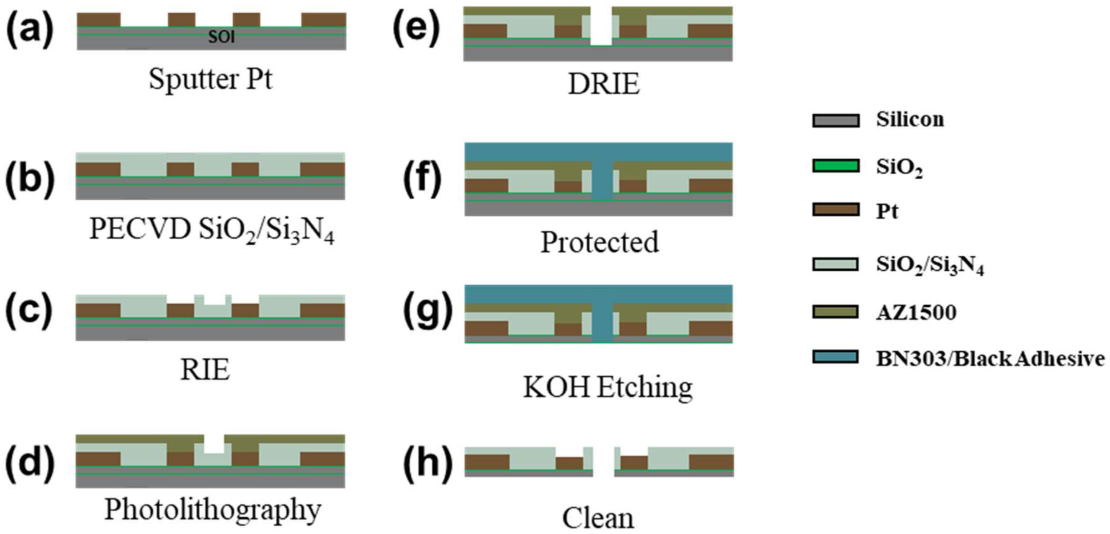

Figure S1 The fabrication process of MEA. (**a**) Ti/Pt were sputtered onto the SOI wafer following lift-off. (**b**) SiO_2/Si_3N_4 were deposited using PECVD. (**c**) The sensitive electrodes were opened by RIE. (**d**) Photoresist used as mask for individual MEA separation. (**e**) Frontal silicon was etched by DRIE. (**f**) BN303 and black adhesive was spin-coated to protect the frontal pattern. (**g**) The back silicon was etched by KOH. (**h**) The individual MEA was released from wafer and cleaned by acetone.

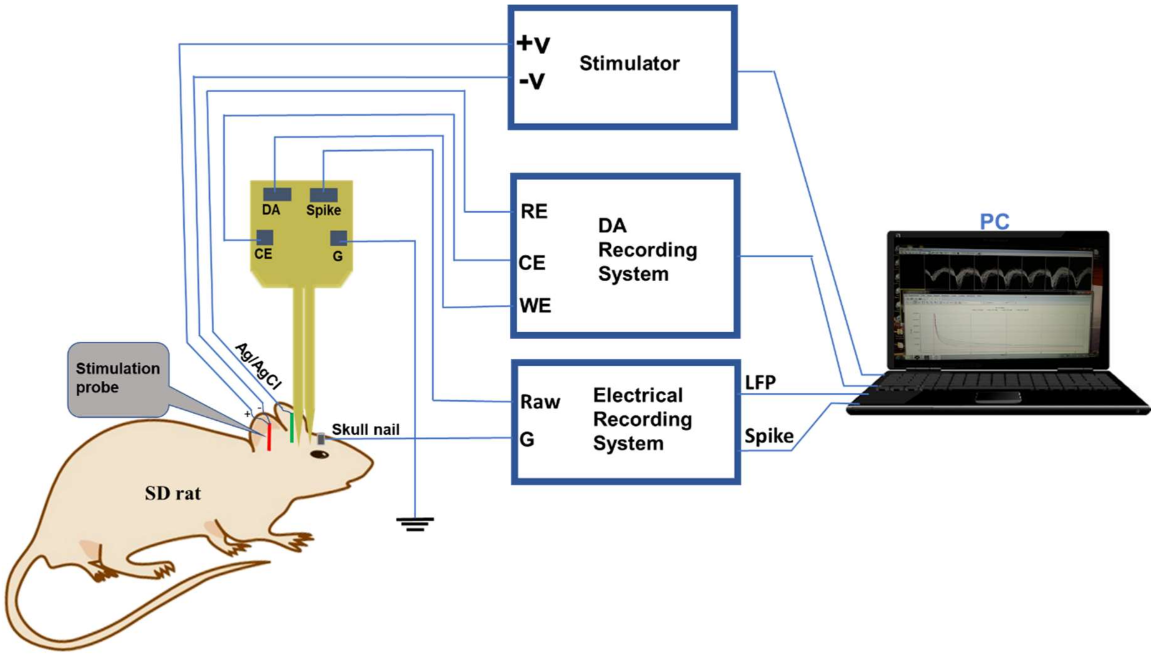

Figure S2. The schematic diagram of detection system including DA recording and electrical recording under stimulation. MEA(yellow), stimulation probe (red), Ag/AgCl reference electrode (green), and skull nail (grey) are implanted in the specific areas as described in the manuscript.

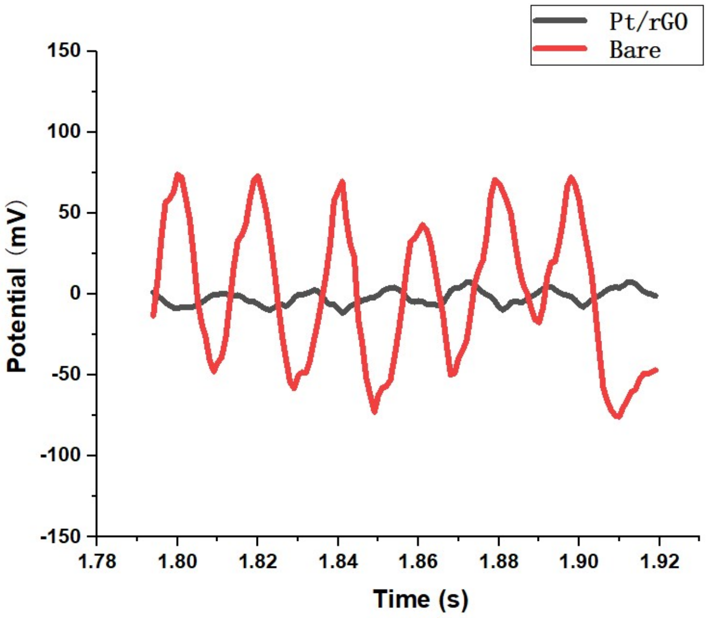

Figure S3. Background noise comparison before and after modification of Pt/rGO.

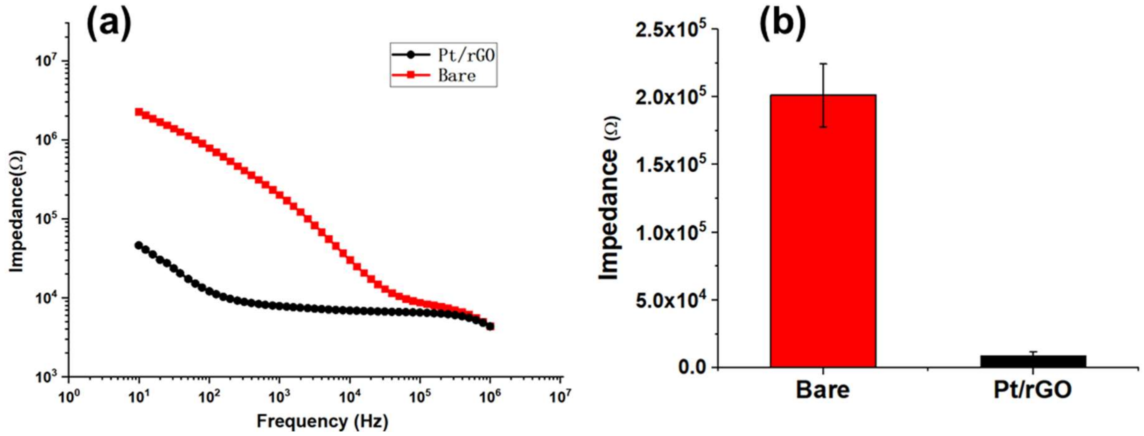

Figure S4. Impedance comparion before and after modification of Pt/rGO. (**a**) Bode plot (impedance-frequency plot) comparison of |Z| values between bare and Pt/rGO electrode. (**b**) The mean impedance comparison at 1 kHz between bare and Pt/rGO electrode.
